# Lower Prevalence of Carotid Plaque Hemorrhage in Women, and Its Mediator Effect on Sex Differences in Recurrent Cerebrovascular Events

**DOI:** 10.1371/journal.pone.0047319

**Published:** 2012-10-26

**Authors:** Neghal Kandiyil, Nishath Altaf, Akram A. Hosseini, Shane T. MacSweeney, Dorothee P. Auer

**Affiliations:** 1 Division of Radiological and Imaging Sciences, University of Nottingham, Nottingham, United Kingdom; 2 Vascular Surgery, Nottingham University Hospitals, Nottingham, United Kingdom; Indiana University School of Medicine, United States of America

## Abstract

**Background and Purpose:**

Women are at lower risk of stroke, and appear to benefit less from carotid endarterectomy (CEA) than men. We hypothesised that this is due to more benign carotid disease in women mediating a lower risk of recurrent cerebrovascular events. To test this, we investigated sex differences in the prevalence of MRI detectable plaque hemorrhage (MRI PH) as an index of plaque instability, and secondly whether MRI PH mediates sex differences in the rate of cerebrovascular recurrence.

**Methods:**

Prevalence of PH between sexes was analysed in a single centre pooled cohort of 176 patients with recently symptomatic, significant carotid stenosis (106 severe [≥70%], 70 moderate [50–69%]) who underwent prospective carotid MRI scanning for identification of MRI PH. Further, a meta-analysis of published evidence was undertaken. Recurrent events were noted during clinical follow up for survival analysis.

**Results:**

Women with symptomatic carotid stenosis (50%≥) were less likely to have plaque hemorrhage (PH) than men (46% vs. 70%) with an adjusted OR of 0.23 [95% CI 0.10–0.50, P<0.0001] controlling for other known vascular risk factors. This negative association was only significant for the severe stenosis subgroup (adjusted OR 0.18, 95% CI 0.067–0.50) not the moderate degree stenosis. Female sex in this subgroup also predicted a longer time to recurrent cerebral ischemic events (HR 0.38 95% CI 0.15–0.98, P = 0.045). Further addition of MRI PH or smoking abolished the sex effects with only MRI PH exerting a direct effect.

Meta-analysis confirmed a protective effect of female sex on development of PH: unadjusted OR for presence of PH = 0.54 (95% CI 0.45–0.67, p<0.00001).

**Conclusions:**

MRI PH is significantly less prevalent in women. Women with MRI PH and severe stenosis have a similar risk as men for recurrent cerebrovascular events. MRI PH thus allows overcoming the sex bias in selection for CEA.

## Introduction

Stroke is a major cause of mortality and severe disability in adults in developed countries. Survivors of a transient ischemic attack (TIA) or stroke represent a population at increased risk of stroke and up to 30% of all strokes are thought to be recurrent strokes [1]. This population offers a unique chance for secondary prevention by pharmacological, lifestyle intervention and carotid endarterectomy (CEA) [2]. Whilst CEA remains an effective means of stroke prevention; current treatment recommendations for CEA are mainly based on symptom status and degree of stenosis [2], but arguably include a large proportion of patients who may not require CEA.

Women are less likely to have carotid disease, and those with significant stenosis are less likely to develop ischemic stroke, and to benefit less from CEA than men [3], [4]. The NASCET and ECST guidelines recommend CEA's in women with carotid artery stenosis over 70%. But it is estimated that patients who have symptomatic carotid artery stenosis greater than 50%, the number of CEA's needed to prevent one disabling stroke is four times higher in women compared to men (NNT are 36 CEA's in women and 9 in men ) [5]. A previously proposed increased operative risk in women [6] has not been confirmed by multivariate analyses of the European Carotid Surgery Trial' (ECST) [7]. More likely, a more benign natural disease progression with faster plaque healing accounts for the lower risk of stroke and hence reduced benefit in women [8], [9]. This poses a clinical dilemma for selecting women for CEA as none of the trials was powered for subgroup analysis, but equally there is substantive doubt as to whether the results derived from predominantly male populations can be applied to female patients. Despite lack of coherent guidelines for women, in clinical practice there seems to be a sex bias in selecting fewer women for carotid endarterectomy than men [10], [11]. In the absence of randomized-control clinical trial evidence for women, there is a pressing need for rational selection criteria in female patients. To address this, a plausible biomarker has to be identified that predicts recurrent risk of stroke in carotid artery disease independent of sex.

Plaque hemorrhage (PH) as detected by MRI [12] may serve as such a biomarker. MRI PH accurately predicts the complex carotid plaque [13]. A number of studies suggested male predominance of more aggressive plaque features with higher prevalence of PH in men [14], [15], [16], [17], [18], [19], [20]. There are however notable discrepancies in the literature with other studies failing to observe a sex difference in prevalence of PH [21], [22], [23]. The reasons for this controversy are unclear but may relate to differences in MRI technique or patient populations. Importantly, our previous findings of more common MRI PH in male patients with symptomatic severe stenosis was based on univariate analysis and may thus have been confounded by other risk factors [19]. Differences in prevalence of PH are potentially clinically relevant as PH was found to predict recurrent ischemic events in symptomatic patients with moderate and severe carotid stenosis [15], [19].

We hypothesized that female sex is independently associated with lower prevalence of MRI PH, and that the lower prevalence may mediate the lower prevalence of recurrent events in women.

The aims of the study were (i) to assess whether women have a lower prevalence of PH independent of other vascular risk factors. To address this, we studied sex differences in a pooled cohort study in symptomatic patients with ≥50% carotid artery stenosis, and undertook a meta-analysis of histological and MRI studies of PH in symptomatic patients. The second aim was (ii) to assess whether MRI PH may mediate potential sex differences in rates of recurrent events. This was pursued by a clinical follow-up sub study in patients with severe stenosis to investigate whether sex differentially affected recurrence rates, and if so whether this was mediated by plaque hemorrhage (PH) or smoking. Smoking is an alternative plausible cause for sex related differences in recurrence rates, as smoking is a proven risk factor for stroke, and there is good epidemiological evidence of its lower prevalence in women [24].

## Methods

A single centre retrospective analysis of research carotid MRI scans drawn from three individual prospective studies, performed with extended clinical follow up.

All individual studies were prospective, required written informed consent, and the pooled analysis was approved by the Nottingham Research Ethics Committee 2.

Participants had been consecutively recruited from the local TIA/Stroke clinic. They were reviewed by either a stroke physician, vascular surgeon or neurology physician with interest in stroke; baseline risk factors and antiplatelet medication post event were recorded (Table 1). To be included in the study, all patients had to have a history of ipsilateral anterior circulation events (Ischemic stroke, Transient Ischemic attack [TIA], or Amaurosis fugax [AmF] and carotid stenosis between 50% to 99% using established Duplex scanning criteria [25]. MRI was conducted as soon as logistically feasible after participants were recruited (median 32 days, range 0–174). Our recruitment rate was approximately 86% as estimated from an audit over a 4 month period from our fast track assessment clinic which took all stroke/TIA referrals from the Nottingham area [26].

**Table 1:** Demographics and risk factors for participants.

	Men (n = 124)	Women (n = 52)	P Value
Age (mean +/−SD)	70+/−10	75+/−8	*0.002
Ischemic heart disease n, (%)	35 (28)	13 (25)	0.66
Diabetes Mellitus n, (%)	15 (12)	2 (4)	0.09
Normotensive n, (%)	26 (21)	10 (19)	
Normotensive with Meds (%)	90 (73)	41 (79)	0.42
‡Hypertensive	8 (7)	1 (2)	
Statin use, <6 M§ before scan (%)	73 (59)	34 (65)	
Statin, >6 M§ before scan	23 (19)	7 (21)	0.66
No statin	28 (23)	11 (14)	
No anticoagulation n, (%)	2 (1.8)	1 (2.2)	
Asprin.	72 (60)	29 (56)	
Clopidogrel.	8 (7)	3 (6)	
Aspirin and Clopidogrel	4 (3)	0 (0)	0.42
Aspirin and Dipyridamole	29 (24)	14 (27)	
Warfarin	4 (3)	5 (10)	
None	4 (3)	1 (2)	
Smokers n,(%)	87 (70)	19 (37)	
†Non-smokers	37 (30)	33 (63)	*<0.0001
Presenting symptoms n, (%)			
Stroke	43 (35)	11 (21)	
TIA	58 (47)	29 (56)	0.20
Amaurosis Fugax	23 (19)	12 (23)	
Time(D§)from presenting symptom to MRI(Mean+/−SD)	462 (907)	364 (768)	0.49
Ipsilateral Degree of stenosis n, (%)			
50–69%	57 (46)	13 (25)	*0.010
70–99%	67 (54)	39 (75)	

* Significantly different (P<0.05) between men and women.

† Non-smokers were defined as having never smoked or stopped smoking for more than 10 years.

‡ Hypertension was defined as by NICE guidelines.

§ M: Months, D:days.

The clinical follow-up data were limited to patients with severe (≥70%, n = 106) carotid stenosis as only in this subgroup a significant and independent association of sex and MRI PH could be established. All study participants were asked to return to a fast access one stop vascular clinic' if they experienced a recurrent cerebrovascular event. Recurrent cerebrovascular events were recorded after MRI in all patients from vascular clinics to CEA, time to recurrent symptoms, death or end of study. All events were verified by a trained neurology research fellow (AH) based on case note review (median follow up 13 days, range 0–1267 days).

### MRI protocol

MRI scans were performed on one of three 1.5-T scanners: Vision, (Siemens Medical), Intera (Philips), or Signa (General Electric) using standard receive-only quadrature neck array coils. A coronal T1-weighted 3-dimensional gradient echo sequence was deployed with blood nulling and effective fat suppression through selective water excitation. This was acquired in a coronal plane (TR 5.8 ms, TE 1.5 ms, FA 15 degrees, TI 19 ms, interpolated voxel size 0.66×0.66×1 mm, acquisition matrix 256×256 (interpolated to 512×512) with 100% rectangular FOV, 72 partitions, acquisition time 251 s).

### MRI analysis

**Figure 1:**
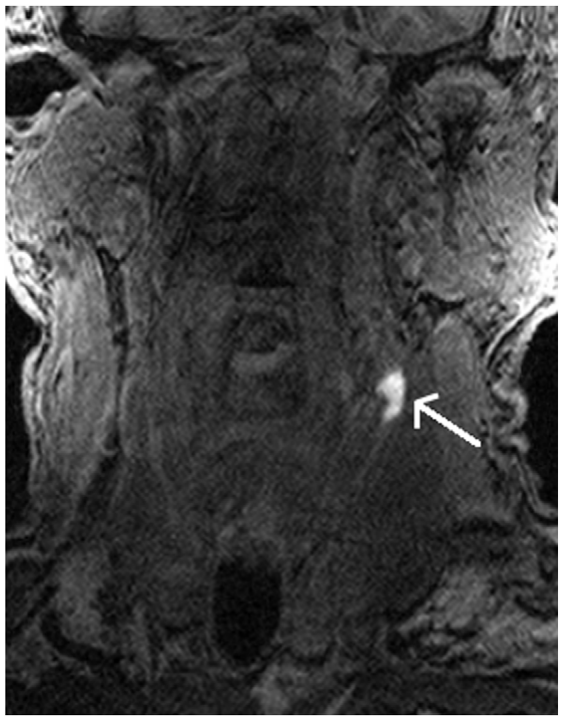
MRI scan showing the presence of carotid plaque hemorrhage seen in a coronal Section, (white arrow).

Presence of plaque hemorrhage [PH] on the MRI scan (MRI PH) was determined by consensus between two blinded experienced researchers based on plaque signal intensity relative to adjacent skeletal muscle by more than 150% (Figure 1).

The cut-off of over 1.5 proved successful in predicting complex carotid plaques on histology using a previously reported MRI and histology dataset [13] and was locally derived using ROC analysis (Altaf N PhD Thesis, University of Nottingham 2008). Ratios of 1.5 or more were categorized as MRI PH +ve and all others MRI PH −ve (see Supplement S1).

### Meta-analysis

A systematic review and meta-analysis was undertaken of papers that analyzed the prevalence of PH in men and women in symptomatic carotid plaques. Two individual researchers (NK & NA) searched the literature to identify 392 (Pubmed) and 401 (Embase) abstracts using the search term of ‘Carotid plaque hemorrhage’. 215 papers were reviewed to assess whether prevalence of plaque hemorrhage were reported between sexes. To avoid duplication, two articles were excluded as it was part of our combined cohort of patients. One study was excluded as it did not contain raw data. One large histological study was retained (Derksen et al.) despite inclusion of asymptomatic patients for its high ratio of symptomatic carotids (84%). Reports on asymptomatic carotid plaque hemorrhage studies were excluded. In total 7 papers met the criteria for the meta-analysis. Meta-analyses were done using RevMan5 software by use of a random effect model.

### Statistical Analysis

All statistical tests were done using SPSS for Windows (version 16.0). Chi-square (χ^2^) and t-tests were used to compare sex difference with categorical, continuous demographic data and risk factors of all patients. Descriptive analysis was used to explore sex differences in the rate of all recurrent ischemic symptoms.

Multivariate logistic regression was used to test for a significant sex difference in PH prevalence independent from known demographic and vascular risk factors.

Kaplan-Meier analysis was used to assess sex effects and MRI PH +ve patients on recurrent ischemic events in patients with severe (70%≥) stenosis. Multivariate Cox regression survival analysis was used to assess sex effects on recurrent ischemic events in patients with severe (≥70%) stenosis controlling for time from symptom to MRI and age with and without addition of the two potential mediator variables, MRI PH and smoking.

The normally distributed data have been displayed as mean +/− standard deviation (SD). The non-normally distributed data have been displayed as median and range.

## Results

176 patients (124 men, 50%≥stenosis) were included. Women were significantly older and had more severe carotid stenosis, whilst men were more frequently smokers than women (Table 1). There were no sex differences in the type of cerebrovascular event or time from ischemic event to MRI scan.

PH in the ipsilateral carotid was seen in 111 patients, and was less prevalent in women (24/52 [46%]) than in men (87/124 [70%], P = 0.003). The difference was also seen for the subgroup with high grade 70–99% stenosis (F: 18/39 [46%] vs. M: 48/67 [72%]; P = 0.009), but failed to reach significance in the moderate stenosis group, 50–69% (F: 6/13 [46%] vs. M: 39/57 [68%], P = 0.131). MRI PH remained significantly less common in women when controlling for age, smoking, ischemic heart disease, and diabetes, (F/M: 24/87 [22% of PH +ve] and 28/37 [43% of PH −ve], adjusted OR 0.23, 95% CI 0.10–0.50, P<0.0001). This association was also found to be significant for the subgroup with high grade stenosis (F/M 18/48 [27% of PH +ve] vs. 21/19 [52% of PH −ve], adjusted OR 0.18, 95% CI 0.07–0.50, P = 0.001). A similar non significant tendency was noted for moderate stenosis alone (F/M 6/39 [13% of PH +ve] vs. 7/8 [47% of PH−ve], adjusted OR 0.25, 95% CI 0.06–1.0, P = 0.06). Also, time from first cerebrovascular event to MRI and anti-platelet medication did not significantly affect these findings.

**Figure 2:**
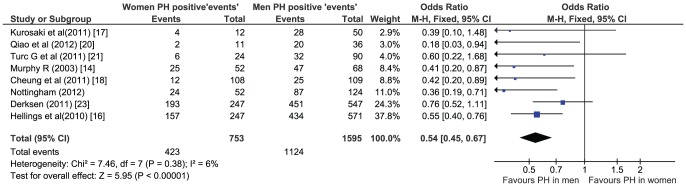
Meta-analysis of our data with previous MRI and histological studies in men and women with symptomatic carotid PH.

Meta-analysis of all histological and MRI studies reporting sex-specific prevalence of PH in symptomatic patients (total number of patients 2348) confirmed lower prevalence of PH in women compared to men (F/M 423/1124 [38%] in PH+ vs. 330/471 [70%] PH−ve, unadjusted OR 0.54, 95% CI 0.45–0.67, P<0.00001). Despite the inclusion of histological studies and heterogeneity of MRI study technique, there was no significant heterogeneity between studies (P = 0.38), Figure 2.

To assess whether such differences in PH prevalence may mediate reported sex differences in recurrent events, we hence limited the analysis to the subgroup of high grade stenosis (70% and above) where we found a significant sex effect of PH prevalence. In this subgroup, a total of 26 recurrent ischemic events (ischemic stroke 7, TIA 14, AmF 5) were recorded during follow up from MRI (median 13 days; range 0–1267 days). 20 of these occurred in male patients (20/67 [30%]) compared to 6 in female patients (6/39 [15%]). When we stratified for MRI PH status, rates of recurrent events became similar between men and women: in presence of MRI PH 33% of women (6/18) and 40% of men (19/48) developed recurrent symptoms, whereas no woman and one man without MRI PH developed recurrent symptoms during follow-up.

**Figure 3:**
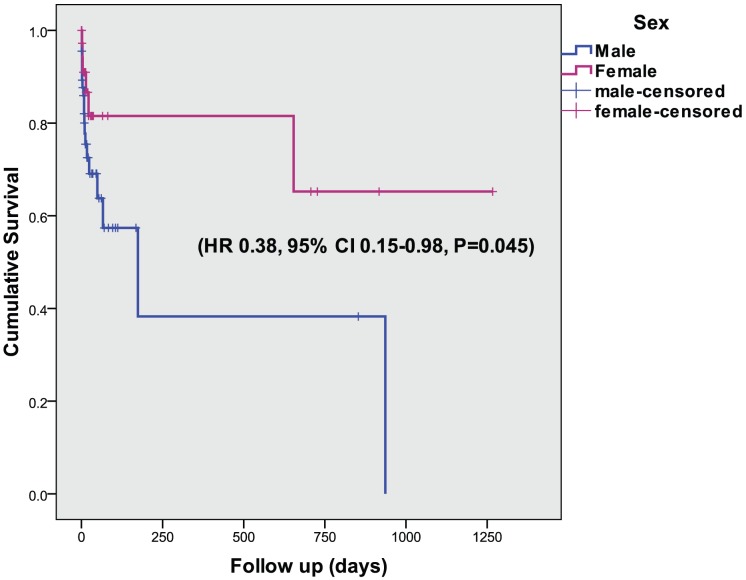
Cumulative survival of all recurrent cerebral ischemic events in men and women with high grade stenosis (70–99%).

**Figure 4:**
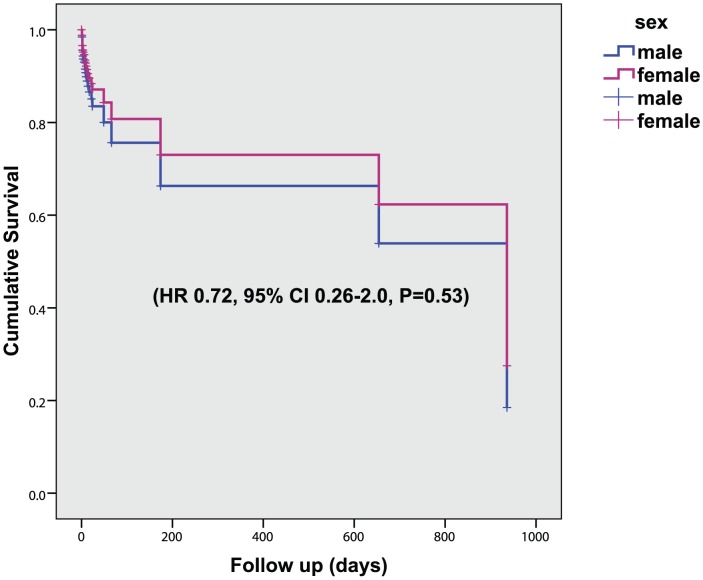
Cumulative survival of all recurrent cerebral ischemic events in men and women with MRI PH +ve high grade stenosis (70–99%).

We formally tested this association and potential mediation using Cox regression survival analysis. A significant protective effect of female sex was seen on all recurrent ischemic events controlling for the time from symptom to MRI and age (HR 0.38, 95% CI 0.15–0.98, P = 0.045), figure 3. Adding MRI PH as additional predictor abolished the sex effect and proved a strong direct effect on recurrent events (HR 22, 95% CI 2.6–157; P = 0.004). Kaplan Meier survival plots confirmed no sex differences for the PH +ve subgroup, figure 4. Smoking, an alternative plausible mediator of the sex effect, also abolished this effect, but failed to reach significance as direct effect on recurrent events (HR 0.41, 95% CI 0.15–1.11; P = 0.081) excluding a strong mediation effect.

## Discussion

Women with severe symptomatic carotid stenosis were found to have a significantly lower prevalence of plaque hemorrhage than men, independent of other major vascular risk factors. Women with ≥70% stenosis were at lower risk of recurrent ischemic events than men, but this difference was found to be at least partially mediated by the lower prevalence of MRI PH.

We found an estimated twofold lower prevalence of MRI PH in female patients with symptomatic carotid stenosis of ≥50% with an estimated fourfold reduction when adjusting for other known vascular risk factors. Our findings are generally well in line with a large histological study [16] and several MRI studies [14], [15], [17], [18], [19], [20] but conflict with other reports [21], [22], [23]. This may be explained by true differences between studied populations or technical differences in determining MRI PH. Hence we conducted a meta-analysis which confirmed an approximately twofold higher prevalence of PH in men. Moreover, we did not observe significant heterogeneity between studies suggesting no substantial effect from different techniques. Some negative studies may have been underpowered. Interestingly, MRI PH studies that did not show significant sex difference reported generally much lower PH prevalence compared to the histological studies [16], [23], [27]. Indeed, a likely type II error may also explain the lack in our study to demonstrate male predominance of PH in the moderate stenosis subgroup. This subgroup contained fewer PH+ patients and only revealed a similar trend. The nature of this sex difference remains unclear, but may relate to more efficient repair as more fibrous or fibroatheromatous plaques are found in women in histology studies [28]. Also carotid plaques appear to heal faster in women than in men which may explain why the benefit from CEA decreases rapidly in women after 2 weeks [5].

We found a lower incidence of any recurrent ischemic symptom in women with 15% of compared to 30% of men experiencing a recurrent ipsilateral AmF, TIA or stroke during clinical follow-up. This is well in line with findings in a larger group of patients with similar symptomatic carotid disease, where the five year cumulative risk of stroke was significantly lower in women [5]. Importantly, when we stratified the groups according to MRI PH status, incidence rates of recurrent events became similar for PH+ men and women, and for PH- men and women suggesting that the lower PH prevalence in women may mediate their observed lower recurrent event rate compared to men.

To test a possible mediation effect of PH, we undertook repeated multivariate Cox regression survival analysis that was limited to the subgroup of patients with severe carotid disease for whom we could establish lower prevalence of PH in women compared to men. We found female sex predicts a longer event-free survival time compared to men controlling for time from initial symptom to MRI and age. This effect was abolished when adding MRI PH, itself a significant predictor of shorter event-free survival in the model. Thus, prevalence of carotid plaque hemorrhage can be inferred to mediate at least partially the sex differences in the risk of recurrent ischemic events.

Sex differences in the prevalence of PH may not be the only mediating factor of lower recurrence risk in women. In fact, at the time of presentation, women were older, had higher degrees of stenosis and were less likely to smoke than men. Amongst these factors, only lower rates of smoking could plausibly exert a mediation effect on lower risk of recurrent events. Smoking is an established vascular risk factor and there is good epidemiological evidence for lower prevalence of smoking in older women [24]. Indeed, adding smoking in the multivariate regression model abolished the sex effect on event-free survival, but smoking status itself failed to reach significance (p = 0.08) in predicting recurrent events. This does not allow ruling out a partial mediation effect from smoking; nevertheless, if contributing this would be a weak affect that is also clinically irrelevant as encouragement to discontinue smoking will be offered to all patients.

There are several limitations to this study; the study was a pooled retrospective analysis across several studies. Nevertheless, individual studies were prospective, consecutive and with high recruitment rates. In addition, standardised protocols were deployed and we are not aware of conceivable potential for bias between sexes. The small number of observed events limited the power of the study and the negative result for smoking has to be cautiously interpreted. The follow-up period was variable and relatively short as delay to CEA was only determined by clinical considerations and logistical constraints of clinical services. These factors are, however, unlikely to have introduced a bias and in fact we did not observe significant sex differences in time from symptom and follow-up period.

We used a single MRI contrast as marker of PH for this study and found 64% prevalence of PH. This is higher than previously reported from multiple MRI sequences, but in very good agreement with large histological series [16], [23], [27] confirming the previously reported high accuracy of this MRI technique [13].

## Conclusion

We demonstrate a significantly lower prevalence of MRI PH in women compared with men with symptomatic severe carotid disease which was independent of other risk factors and confirmed an overall male predominance for PH in symptomatic patients by a meta-analysis. Moreover, the lower prevalence of MRI PH in woman was shown to mediate a lower risk of recurrence events. In line with our hypothesis we conclude that presence of MRI PH defines a high recurrence risk independent of sex. This may assist selection of female patients with otherwise unclear indication for CEA, such as those patients presenting beyond two weeks from their initial symptom.

## Supporting Information

Supplement S1 **MRI analysis.**
(DOCX)
