# Supplementary material for: Lower Prevalence of Carotid Plaque Hemorrhage in Women, and Its Mediator Effect on Sex Differences in Recurrent Cerebrovascular Events
Source: PLoS One. 2012 Oct 26;7(10):e47319. doi: 10.1371/journal.pone.0047319 (PMC3482217; doi:10.1371/journal.pone.0047319)
Supplement: Supplement S1 — MRI analysis. (DOCX) [file pone.0047319.s001.docx]

**Supplement: MRI analysis (S1)**

Quantitative image analysis was performed off-line using of coronal and axial source data together with standard image reconstruction techniques as provided by using JAVA Imaging (JIM) software ([www.xinapse.com](http://www.xinapse.com)). Using the JIM image analysis program, all areas of ‘bright’ signal within the carotid plaque, suggesting hemorrhage were identified and a region of interest (ROI) of this apparent plaque hyperintensity (HI) was drawn over each area without selecting the edge detection tool. This was performed using the threshold, irregular ROI option in JIM. The mean intensity of the apparent plaque HI was calculated (Icar). Next, the control ROI was selected and placed over the adjacent sternocleidomastoid muscle aiming to keep at equidistance from the surface coil. The mean intensity within the averaged ROI over the muscle (Imus) and plaque HI was computed. The ratio of the intensity of the carotid plaque over the muscle was calculated in axial and coronal plane (Icar /Imus). If consistent ratios of ≥1.5 were determined the plaque was considered PH+ve.

If no subjective hyperintensity was seen, a second observer qualitatively assessed presence or absence of HI, and furthermore signal intensities were quantified by placing a ROI in the wall of the carotid plaque closest to carotid bifurcation with similar process for control ROI in the adjacent muscle. If SI was <1.5, the plaque was defined PH-ve.

There was very good intra-rater agreement reliability for the intensity ratio’s (Icar /Imus) (Kappa value (k) =0.80), with also very good equivalent intra-rater and inter-rater reliability agreement (k=0.78) for the presence and absence of PH.
